# International medical students’ acculturation and self-rated health status in Hungary: a cross-sectional study

**DOI:** 10.1186/s12889-022-14334-y

**Published:** 2022-10-19

**Authors:** Afriza Umami, Edit Paulik, Regina Molnár

**Affiliations:** 1 Department of Public Health, Albert Szent-Györgyi Medical School, University of Szeged, Dóm tér 10, H- 6720 Szeged, Hungary; 2 Stikes Muhammadiyah Bojonegoro, Bojonegoro, Indonesia

**Keywords:** Acculturation, General health, International medical students, Mental health, Self-rated health

## Abstract

**Background:**

Over the past few decades, the number of international students has increased dramatically. These students have to adjust to unfamiliar social, cultural, and educational environments. The concept of acculturation has been applied in multiple studies on various health outcomes. This study investigated the relationship between acculturation and self-rated health (SRH) among international medical students.

**Methods:**

A cross-sectional study was conducted among international medical students at the University of Szeged, Hungary between April and October 2021. A total of 326 participants filled out questionnaires about sociodemographic characteristics, acculturation, and SRH. The modified Stephenson multigroup acculturation scale (SMAS) was used to assess the acculturation status; the scale defined acculturation as the degree of dominant society immersion (DSI, 12 items) and ethnic society immersion (ESI, 16 items). To measure SRH, participants were asked to rate their current general health and mental health. The data were analyzed by using descriptive statistics and the multiple logistic regression model.

**Results:**

32.5% of the students reported having poor general and 49.7% poor mental health. We have found that acculturation was associated with SRH in multivariable logistic regression models controlling for sociodemographic characteristic. Bidimensional acculturation, such as ESI and DSI significantly influenced SRH as the likelihood of poor general health decreased (OR = 0.50; 95% CI = 0.31–0.81, P = 0.005), when the ESI was higher, whereas the likelihood of poor mental health decreased (OR = 0.52; 95% CI = 0.35–0.79, P = 0.002) if students had a greater DSI.

**Conclusion:**

Both types of immersion can affect the students’ SRH. If the student could integrate better into their own ethnic group, their general health was better, and if they could strongly integrate into the Hungarian society, their mental health was more favorable. Acculturation measures should be promoted by academics and public health professionals in order to better understand their role in the behaviors, health outcomes, and health care use of medical international students. These findings will help professionals shape culturally sensitive prevention and counselling strategies for international student populations.

**Supplementary Information:**

The online version contains supplementary material available at 10.1186/s12889-022-14334-y.

## Background

Studying abroad has become commonplace, and universities around the world welcome large numbers of international students [1]. There were 1.3 million students from abroad who were undertaking tertiary level studies across the European Union (EU) in 2018 [2]. As a member of the EU, Hungary is one of the educational destinations for international students at 11.4% [2] Over 32 thousand international students were enrolled at Hungarian universities in the academic year of 2020/2021 [3]. About one-third of the foreign students go to one of the four Hungarian medical universities [4]. It is very popular with medical students due to the growing demand for migrant healthcare workers. Similarly, the limited share of medical schools in their home countries encourages students to continue their medical education abroad [5]. International medical students (IMSs) migrate abroad solely for the purpose of studying [6].

However, the decision to study abroad in Western countries may present several challenges to international students, including the acculturative stress and difficulties adjusting to the host country’s environment [6], they live far from their family and have to find new friends, there are new types of foods to get accustomed to, and new learning and communication styles. They must accept the local customs and traditions, often experiencing cultural pressure or even culture shock, which is known as acculturation, a dynamic, complex, and multidimensional process of adaptation [7]. Acculturation is not the same for all international students. Students’ degrees of acculturation towards other cultures vary depending on their different backgrounds and other factors, resulting in different preferences for which acculturation strategies to employ [8].

Many researchers see acculturation as a single continuum-changing process, moving from maintenance of the hometown culture on one side to immersion in the host culture on the other [9], generating the unidimensional model. However, for the past few decades, viewing acculturation as a unidirectional process has been critiqued as prohibiting migrants from acculturation to both the host as well as the origin culture simultaneously [10]. The new theoretical framework argues that the maintenance of the hometown culture and the adoption of the host culture must be seen as two independent dimensions [7, 8]. The new bidimensional model was proposed suggesting that the increase or decline of one culture does not affect the other. Most studies have used Berry’s four-cell typology model (assimilation, separation, integration, and marginalization), which adhered to the bidimensional theory [11]. The different acculturations depend on the individual’s desire to take part in the host culture as well as the cultural attitude of the receiving society [12].

The relationship between acculturation and health has attracted general attention [13, 14]; however, the framework theory of acculturation is still surrounded by controversy. Within the various theoretical frameworks, the relationship between acculturation and health has been studied for decades [15–17]. Acculturation has been proven to influence health through healthy behaviors, access to healthcare services, social support, self-esteem, and stress [12, 18, 19]. Most empirical studies using a unidimensional model have consistently found that non-dominant groups with low acculturation are more likely to have general health issues [20] and diabetes [21, 22]. However, studies have also revealed that the relationship between acculturation and health is not linear [20].

Since the outbreak of COVID-19 in December 2019 it has spread rapidly to almost all parts of the world [23], everyone is concerned about health issues, the economy, and the educational system [24]. A large number of students was having difficulty with their academic progress as a result of the closure of the campus and the switch to online learning [25]. According to the Student Covid Insights Survey (SCIS) performed by the Office for National Statistics (ONS) in 2020, over 50% of students in higher education in England experienced negative effects on their mental health and general well-being as a result of the COVID-19 pandemic [26]. According to the SCIS report, students expressed higher levels of anxiety and poorer levels of life satisfaction and happiness during the pandemic than the general population. Additionally, 18% of students had moderate-to-severe insomnia symptoms, and 12% reported moderate-to-severe symptoms of anxiety and depression [27]. These issues are exacerbated by multiple periods of self-isolation that many international students have experienced, which has ultimately been very challenging for their mental well-being [28, 29].

Although studies have assessed how acculturation status predicts the status of various health outcomes, it remains unclear whether acculturation status truly predicts one’s SRH. In the current study, the population investigated is international medical students, and in this population, research related to acculturation and SRH has still rarely been conducted. In the present study, we measured SRH in two different health states: general health status and mental health status. Therefore, the aim of our study was to examine the association between acculturation and self-rated health among international medical students. Moreover, we hypothesized that the level of acculturation was associated with SRH status, and this relationship was confounded by sociodemographic characteristics and ethnic minority.

## Methods

### Study design and participants

In the cross-sectional study, international medical students from University of Szeged were invited to complete an online questionnaire using the online platform Survio (https://www.survio.com/). Data were collected from April to October 2021. We used convenience sampling; all international medical students from first to sixth years were invited to participate. The participation was voluntary and anonymous. Altogether 326 students filled out the questionnaire. The study protocol was approved by the Human Institutional and Regional Biomedical Research Ethics Committee, University of Szeged, Hungary (license number: 4936).

## Variables and measurement

### Dependent variable: self-reported Health (SRH) Status

SRH is one of the most frequently used measures in epidemiological, clinical, and social research [30]. In this study, SRH was measured from the point of general health and mental health reported by using a five-point Likert scale (“very bad = 1”, “bad = 2”, “moderate = 3”, “good = 4”, or “very good = 5”). The respondents had to answer the question regarding general health, “how do you evaluate your general health status?“ And for mental health they were asked, “how do you evaluate your mental health status?“ For the purposes of data analyses, SRH was categorized as good (scales 4 and 5) and poor (scales from 1 to 3).

## Independent variable: acculturation

To assess the acculturation status of international medical students in Hungary, we used the modified Stephenson multigroup acculturation scale (SMAS). The original SMAS is a 32-item questionnaire, initially developed for respondents from five different ethnic groups in America. The scale yields two dimensions: Ethnic society immersion (ESI) and dominant society immersion (DSI). Within both dimensions, there are four domains were assessed: language, interaction, media, and food, and each domain reflects knowledge, behaviors, and attitudes (e.g., language knowledge, language behavior, and language attitude) [31]. The answers included a Likert response format: 1 = false, 2 = partly false, 3 = partly true, and 4 = true. The ESI score reflects the level to which one retains values and practices of an ethnic group (home country), whereas the DSI score reflects the extent to which an individual adopts the practices of the dominant society (host country). This questionnaire was adapted by Asif (2018), in which he modified the original version by removing some questions related to the English language ability of respondents since being able to read, write, and speak English was one of the inclusion criteria [32]. Cronbach’s alpha in Asif’s study was 0.72 for DSI and 0.62 for ESI. In the current study, we used this modified 28-item questionnaire (ESI = 16 items, DSI = 12 items). The Cronbach’s alpha of the modified SMAS in our study was 0.82 for ESI and 0.88 for DSI (see Additional file 1). The summed mean scores for the subscales were used for statistical analysis, with a higher score reflected greater immersion on each dimension of either ESI or DSI. The items of the questionnaire are available in the Supplementary Information (see Additional file 2).

## Covariates

Demographic characteristics: age was measured as a continuous variable (years); gender was categorized as “male” or “female”; relationship status was dichotomized as “not in relationship (single/divorced/living separated)” and “in relationship (married/common-law marriage/living together/having partner but not living together)”; and country of origin was classified according to the continent of the home country as “European” and “non-European” (Africa, America, Asia, and Middle East).

Years of study were categorized as “preclinical (1st /2nd )” and “clinical (3rd /4th /5th /6th )”. The economic condition of the students’ family was evaluated by a 5-point Likert scale, and it was dichotomized as “low income” (very bad/bad/average)” and “high income (good/very good)”. Ethnic minority, whether students belong to an ethnic minority in their home country, had “no” or “yes” answers.

### Statistical analysis

We used descriptive statistics to summarize the sample characteristics including frequency, percentage, mean, and standard deviation (SD). Univariable binary logistic regression was used to assess the unadjusted odd ratio between dependent and independent variables. The dependent variables were self-rated general health and self-rated mental health. Multivariable logistic regression analyses were performed to examine the relationship between acculturation and self-rated health by adjusting for covariates. The independent variable involved in the regression analysis was acculturation: ESI and DSI, while covariates were student age, gender, year of study, country of origin, relationship status, economic status, and ethnic minority. Student age, ESI, and DSI were considered as continuous variables in the model. The Hosmer–Lemeshow test was used to determine the goodness of fit of the logistic regression model. Odds ratio (OR) and 95% confidence interval (CI) were used to indicate the association between acculturation and independent variables. Statistical significance was defined at p < 0.05.

Data analysis was carried out with IBM SPSS (Statistical Package for the Social Sciences) version 27 (SPSS Inc., Chicago, IL, USA).

## Results

Descriptive characteristics of the participants are presented in Table 1. The average age was 22.86 ± 2.86 years. 53.7% of the students were male, and more than half were in the preclinical years of medical school. More participants reported higher economic family income and no ethnic minority status. The majority of students were from non-European countries (34.7% of the participants were from the Middle East, 19.0% were Asians, 5.2% were Americans, and 1.8% were Africans). More than half of medical international students reported good general health status (67.5%), while half of them reported good mental health status (50.3%).

The current study measured acculturation along two dimensions: the ESI score is 3.19 ± 0.47, which reflects the extent to which an individual retains the values and practices of an ethnic group (home country), and the DSI score is 2.05 ± 0.57, which reflects the extent to which an individual adopts the practices of the dominant society (host country). Most international students still prefer to speak in their native language and read and write in their native language in the ESI dimension. While on the DSI dimension, most students felt at home in Hungary, got information about current affairs in Hungary, and felt accepted by Hungarians. However, their understanding of the Hungarian language was inadequate. The distribution of the answers is available in Supplementary Information (see Additional file 2).

**Table 1:** Characteristics of respondents (N = 326)

Characteristic	Category	Mean ± SD	N	(%)
Age (year)		22.86 ± 2.86		
Gender	Male		151	46.3
	Female		175	53.7
Year of study	Preclinical		224	68.7
	Clinical		102	31.3
Country of origin	Non-European		198	60.7
	European		128	39.3
Relationship status	Not in a relationship		224	74.8
	In a relationship		82	25.2
Economic status	Low income		86	26.4
	High income		240	73.6
Ethnic minority	No		278	85.3
	Yes		48	14.7
Self-rated general health	Good		220	67.5
	Poor		106	32.5
Self-rated mental health	Good		164	50.3
	Poor		162	49.7
Acculturation	ESI	3.19 ± 0.47		
	DSI	2.05 ± 0.57		

ESI = Ethnic Society Immersion; DSI = Dominant Society Immersion; SD = standard deviations

The descriptive analyses of general health and mental health are presented in Table 2.

**Table 2:** Cross tabulations of self-rated general health and self-rated mental health

	General health				Mental health					
Variables	Good (220)		Poor (106)		Good (164)			Poor (162)		
	n	%	n	%	n		%	n	%	
**ESI (mean ± SD)**	(3.25 ± 0.42)		(3.09 ± 0.56)		(3.26 ± 0.39)			(3.13 ± 0.53)		
**DSI (mean ± SD)**	(2.10 ± 0.55)		(1.95 ± 0.58)		(2.15 ± 0.58)			(1.95 ± 0.53)		
**Age (mean ± SD)**	(22.71 ± 2.88)		(23.16 ± 2.79)		(23.02 ± 3.04)			(22.69 ± 2.65)		
**Gender**										
Male	108	33.1	43	13.2	79	24.2		72		22.1
Female	112	34.4	63	19.3	85	26.1		90		27.6
**Year of study**										
Preclinical	156	47.9	68	20.9	112	34.4		112		34.4
Clinical	64	19.6	38	11.7	52	16.0		50		15.3
**Country of origin**										
Non-European	127	39.0	71	21.8	96	29.4		102		31.3
European	93	28.5	35	10.7	68	20.9		60		18.4
**Relationship status**										
Not in a relationship	165	50.6	79	24.2	125	38.3		119		36.5
In relationship	55	16.9	27	8.3	39	12.0		43		13.2
**Economic status**										
Low income	53	16.3	33	10.1	40	12.3		46		14.1
High income	167	51.2	73	22.4	124	38.0		116		35.6
**Ethnic minority**										
No	190	58.3	88	27.0	147	45.1		131		40.2
Yes	30	9.2	18	5.5	17	5.2		31		9.5

ESI = Ethnic Society Immersion; DSI = Dominant Society Immersion; SD = standard deviations

Table 3 shows the univariable and multivariable logistic regression analyses of self-rated general health. According to the univariable regression analysis, ESI and DSI were significantly associated with general health status. For every unit increase in ESI, the odds of poor general health decreased by 50% (Unadjusted Odds Ratio (UAOR) = 0.50; 95% CI = 0.31–0.81, P = 0.005) and each 1-unit increase in DSI reduced the odds of poor general health (UAOR = 0.60; 95% CI = 0.39–0.94, P = 0.024). According to the multivariable logistic regression model, ESI negatively influenced the poor general health of international medical students after adjusting for all covariates. The adjusted odds ratio (AOR) for ESI was 0.51 (95% CI = 0.30–0.87; P = 0.014), indicating that holding all other variables constant in the model, with every unit increase in ESI, the students were 0.51 times less likely to have poor general health.

**Table 3:** Univariable and multivariable logistic regression analysis of sociodemographic, acculturation and self-rated general health (poor general health)

Variables	Poor general health							
	UAOR	95% CI		P-value	AOR	95% CI		P-value
		Lower	Upper			Lower	Upper	
**ESI (mean ± SD)**	0.50	0.31	0.81	**0.005**	0.51	0.30	0.87	**0.014**
**DSI (mean ± SD)**	0.60	0.39	0.94	**0.024**	0.69	0.43	1.11	0.124
**Age (mean** ± SD**)**	1.06	0.98	1.14	0.183	1.08	0.99	1.18	0.103
**Gender**								
Male	Ref	0.89	2.26	0.148	Ref	0.87	2.34	0.155
Female	1.41				1.43			
**Year of study**								
Preclinical	Ref	0.83	2.23	0.218	Ref	0.52	1.70	0.849
Clinical	1.36				0.94			
**Country of origin**								
Non-European	Ref	0.41	1.09	0.109	Ref	0.45	1.40	0.428
European	0.67				0.79			
**Relationship status**								
Not in a relationship	Ref	0.60	1.75	0.927	Ref	0.65	2.08	0.617
In relationship	1.03				1.16			
**Economic status**								
Low income	Ref	0.42	1.17	0.177	Ref	0.46	1.37	0.411
High income	0.70				0.79			
**Ethnic minority**								
No	Ref	0.69	2.45	0.425	Ref	0.55	2.12	0.824
Yes	1.29				1.08			

UAOR: unadjusted odds ratio; AOR: adjusted odds ratio; 95% CI: 95% confidence interval

In case of adjusted model: Hosmer–Lemeshow goodness of fit test χ^2^ = 7.067, df = 8, p = 0.529

Table 4 demonstrates the univariable and multivariable logistic regression analyses of self-rated mental health. The unadjusted results revealed that ESI and DSI were associated with lower poor mental health, while students who had been exposed to ethnic minorities were more likely to have poor metal health than those who have no experienced ethnic minorities (UAOR = 2.05; 95% CI = 1.08–3.87, P = 0.025). According to the results of multiple logistic regression models of mental health, DSI negatively influenced poor mental health of international medical students after adjusting for all covariates (Table 4). The odds ratio for DSI was 0.52 (AOR = 0.52; 95% CI = 0.52 − 0.33; P = 0.004), indicating that holding all other variables constant in the model, with every unit increase in DSI, the students were 0.52 times less likely to have poor mental health.

**Table 4:** Univariable and multivariable logistic regression analysis of sociodemographic, acculturation and self-rated mental health (poor mental health)

Variables	Poor mental health							
	UAOR	95% CI		P	AOR	95% CI		P
		Lower	Upper			Lower	Upper	
**ESI (mean ± SD)**	0.53	0.33	0.87	**0.012**	0.64	0.38	1.08	0.100
**DSI (mean ± SD)**	0.52	0.34	0.78	**0.002**	0.52	0.34	0.82	**0.004**
**Age (mean** ± SD**)**	0.96	0.89	1.04	0.302	0.98	0.89	1.07	0.615
**Gender**								
Male	Ref	0.75	1.80	0.500	Ref	0.68	1.71	0.743
Female	1.16				1.08			
**Year of study**								
Preclinical	Ref	0.60	1.54	0.870	Ref	0.46	1.43	0.474
Clinical	0.96				0.81			
**Country of origin**								
Non-Europe	Ref	0.53	1.30	0.413	Ref	0.59	1.68	0.978
Europe	0.83				0.99			
**Relationship status**								
Not in a relationship	Ref	0.70	1.91	0.565	Ref	0.85	2.57	0.168
In relationship	1.16				1.48			
**Economic status**								
Low income	Ref	0.50	1.33	0.412	Ref	0.47	1.36	0.407
High income	0.81				0.79			
**Ethnic minority**								
No	Ref	1.08	3.87	**0.025**	Ref	0.99	3.87	0.051
Yes	2.05				1.97			

UAOR: unadjusted odds ratio; AOR: adjusted odds ratio; 95% CI: 95% confidence interval

In case of adjusted model: Hosmer–Lemeshow goodness of fit test χ^2^ = 7.038, df = 8, p = 0.533

## Discussion

The aim of this study was to better understand the relationship between acculturation and SRH among international medical students. Specifically, we investigated whether acculturation was related to general and mental health, after controlling for all covariates.

According to the findings of this study, 32.5% of international medical students reported to have poor general health. Meanwhile, nearly half of the students reported to have poor mental health. These research results are consistent with the findings of Ochnik et al. (2021), who have found that the prevalence of mental health problems in university students is high, with the prevalence of high stress, depression, and generalized anxiety symptoms in the total sample being 61.30%, 40.3%, and 30%, respectively [33]. Furthermore, international students reported higher rates of depression, suicidal ideation, anxiety, post-traumatic stress disorder (PTSD), academic stress, and loneliness [34]. Several studies show that tertiary education students have a higher incidence of mental health problems than the general population, and that international students have higher levels of anxiety than domestic students [35].

Our current research divided acculturation into two dimensions: on the one hand, dominant society immersion, in which students can meet and interact with the host country’s society, culture, beliefs, and attitudes, and on the other hand, ethnic society immersion, in which students can maintain cultural values and heritage from their home country.

Our findings suggest that high ethnic society immersion, in which students retain their ethnic group’s values and practices (home country), tends to decrease the poor general health status. This is consistent with the findings of Suleiman et al. (2021), who have discovered that greater heritage identity is associated with a lower risk of poor self-rated health [36]. Meanwhile, research conducted among Asian immigrant groups has found that the separated are significantly more likely than the assimilated to report poor-to-fair SRH [37].

In addition, we discovered that dominant society immersion had no significant relationship with self-rated general health. Meanwhile, Wang’s study describes that acculturation is associated with a higher likelihood of reporting excellent or good health among older Chinese Americans [27]. Other studies have argued that acculturation was a risk factor for SRH [38, 39]; a study conducted by Johnson et al. (2010) has used Andersen’s socio-behavioral approach, which allows for a better understanding of how acculturation affects health status by systematically evaluating a group of determinants and their impact on SRH, and the findings have pointed at that Mexican-oriented acculturation remains an independent predictor of fair/poor health [38].

The current study found that high levels of dominant society immersion was associated with a lower risk of poor mental health. The majority of international students felt accepted by Hungarians and received information about current events in Hungary. This might reduce students’ feelings of loneliness in a new environment.

The results of our study seem to be consistent with the findings of a systematic review, which has discussed that marginalization had the most negative effects on the mental health of migrant populations, while integration had the most positive effects. The study also identifies three major sources of acculturation stress and poor mental health, such as a lack of education or skill set, a lack of proficiency in the host country’s language, and financial difficulties [13].

According to some theories, acculturation can help with everyday social interactions [40, 41]. Nonetheless, acculturation can increase stress or conflict between two competing cultures [42] or be associated with decreased social support [43]. Not surprisingly, empirical findings have been mixed, with some studies linking higher acculturation to a new culture to poorer SRH [38, 39], while others show favorable relationship to mental health [37, 44] or no relationship at all [36].

## Limitations of the study

Several limitations of the current study need to be considered while interpreting the results. The data obtained were based on self-report, which can lead to response bias. Second, the data are cross-sectional in nature, thus preventing the authors from drawing causal conclusions. Self-rated health possibly influences acculturation. Whereas the general health status and low mental health will affect the acculturation process in international students. Third, this study used a non-probability sampling; thus, the sample might not be representative. Fourth, while self-rated health is an informative measure of older adults’ general health status, future studies are encouraged to use objective health measures to enhance rigor. Therefore, future studies should include these indicators in measuring acculturation. Finally, we did not measure acculturation stress in this study, which is an important factor in poor health outcomes and the health effects of COVID-19 pandemic should be considered to the results of the survey.

## Conclusion

This study highlights the relationship between acculturation and self-health assessment by controlling for confounding variables, such as demographics, socioeconomic status and ethnic minorities. Measurement of acculturation uses two dimensions, namely ethnic society and dominant society, which provide a two-way explanation of the concept of acculturation. The likelihood of poor general health decreased when the ESI was high, whereas the DSI had no significance for poor general health. Poor mental health will decrease if students have a greater DSI, while ESI is not associated with the incidence of poor mental health among international students. However, the results also demonstrated that sociodemographic and ethnic minority were unrelated to students’ general health status, while ethnic minority had an influence on their mental health status but not sociodemographic characteristics.

Understanding the relationship of acculturation on the general and mental health status of these special populations is important. Using the international student population to understand the impact of acculturation on health is still a rarity in research; therefore, these results can be used to provide information on improving the health of international students during their university studies. Our findings are intended to inspire future research about the potential psychosocial determinants of self-rated health among international students. Providing culturally sensitive services and finding ways to strengthen and develop social support for international students while facilitating their integration will benefit their general and mental health.

## Electronic supplementary material

Below is the link to the electronic supplementary material.

Supplementary Material 1

Supplementary Material 2

## Data Availability

The datasets used during the current study are available from the corresponding author on reasonable request.

## Abbreviations

DSI: Dominant society immersion.
ESI: Ethnic society immersion.
EU: European Union.
IMSs: International medical students.
PTSD: Post-traumatic stress disorder.
SMAS: Stephenson multigroup acculturation scale.
SRH: Self-rated health.
SCIS: Student Covid Insights Survey.
ONS: Office for National Statistics.
