# Supplementary material for: International medical students’ acculturation and self-rated health status in Hungary: a cross-sectional study
Source: BMC Public Health. 2022 Oct 19;22:1941. doi: 10.1186/s12889-022-14334-y (PMC9580418; doi:10.1186/s12889-022-14334-y)
Supplement: Supplementary file 1 — Supplementary Material 1 [file 12889_2022_14334_MOESM1_ESM.docx]

**Additional file 1**

Reliability statistics of ESI and DSI

**Alpha Cronbach’s ESI**

| **Case Processing Summary** | | | |
| --- | --- | --- | --- |
|  | | N | % |
| Cases | Valid | 15 | 100.0 |
|  | Excluded^a^ | 0 | .0 |
|  | Total | 15 | 100.0 |
| a. Listwise deletion based on all variables in the procedure. | | | |

| **Reliability Statistics** | |
| --- | --- |
| Cronbach's Alpha | N of Items |
| .819 | 16 |

| **Item-Total Statistics** | | | | |
| --- | --- | --- | --- | --- |
|  | Scale Mean if Item Deleted | Scale Variance if Item Deleted | Corrected Item-Total Correlation | Cronbach's Alpha if Item Deleted |
| 1. I speak my native language with my friends and acquaintances from my country of origin. | 49.73 | 45.638 | .509 | .808 |
| 2. I have never learned to speak the language of my native country. | 51.87 | 46.124 | .195 | .825 |
| 3. I eat traditional food from my native culture. | 50.07 | 44.781 | .256 | .823 |
| 4. I feel comfortable speaking my native language. | 49.40 | 47.829 | .416 | .816 |
| 5. I am informed about current affairs in my native country. | 49.67 | 47.952 | .176 | .821 |
| 6. I know how to read and write in my native language. | 49.60 | 45.686 | .283 | .818 |
| 7. I attend social functions with people from my native country. | 49.60 | 44.829 | .528 | .806 |
| 8. I speak my native language at home. | 49.53 | 43.838 | .482 | .806 |
| 9. I regularly read magazines of my ethnic group. | 51.47 | 39.838 | .583 | .797 |
| 10. I know how to speak my native language. | 49.33 | 49.381 | .000 | .823 |
| 11. I am familiar with the history of my native country. | 49.87 | 43.124 | .508 | .804 |
| 12. I like to listen to music of my ethnic group. | 49.87 | 39.552 | .653 | .792 |
| 13. I like to speak my native language. | 49.40 | 47.829 | .416 | .816 |
| 14. I speak my native language with my spouse or partner. | 50.80 | 33.886 | .755 | .781 |
| 15. When I pray, I use my native language. | 50.13 | 38.124 | .618 | .794 |
| 16. I stay in close contact with my family members and relatives in my native country. | 49.67 | 44.381 | .562 | .804 |

**Alpha Cronbach’s DSI**

| **Case Processing Summary** | | | |
| --- | --- | --- | --- |
|  | | N | % |
| Cases | Valid | 15 | 100.0 |
|  | Excluded^a^ | 0 | .0 |
|  | Total | 15 | 100.0 |
| a. Listwise deletion based on all variables in the procedure. | | | |

| **Reliability Statistics** | |
| --- | --- |
| Cronbach's Alpha | N of Items |
| .880 | 12 |

| **Item-Total Statistics** | | | | |
| --- | --- | --- | --- | --- |
|  | Scale Mean if Item Deleted | Scale Variance if Item Deleted | Corrected Item-Total Correlation | Cronbach's Alpha if Item Deleted |
| 1. I am informed about current affairs in Hungary. | 20.67 | 54.095 | .309 | .887 |
| 2. I feel totally confident with Hungarian people. | 20.60 | 50.400 | .534 | .873 |
| 3. I have many Hungarian acquaintances. | 21.33 | 47.381 | .777 | .857 |
| 4. I feel home in Hungary. | 20.73 | 48.781 | .555 | .873 |
| 5. I feel accepted by Hungarians. | 20.47 | 49.695 | .567 | .871 |
| 6. I know how to prepare Hungarian foods. | 21.93 | 50.352 | .759 | .862 |
| 7. I regularly read a Hungarian newspaper. | 22.00 | 51.429 | .598 | .869 |
| 8. I speak Hungarian at home. | 22.13 | 51.695 | .687 | .866 |
| 9. I am familiar with important people in Hungarian history. | 21.53 | 47.124 | .732 | .860 |
| 10. I think in Hungarian. | 22.33 | 59.952 | .000 | .887 |
| 11. I speak Hungarian with my spouse or partner. | 22.00 | 51.714 | .574 | .871 |
| 12. I like to eat Hungarian foods. | 20.93 | 48.495 | .703 | .862 |
