# Supplementary material for: International medical students’ acculturation and self-rated health status in Hungary: a cross-sectional study
Source: BMC Public Health. 2022 Oct 19;22:1941. doi: 10.1186/s12889-022-14334-y (PMC9580418; doi:10.1186/s12889-022-14334-y)
Supplement: Supplementary file 2 — Supplementary Material 2 [file 12889_2022_14334_MOESM2_ESM.docx]

**Additional file 2**

Descriptive Statistics for the Stephenson Multigroup Acculturation Scale (SMAS)

| No | Items | M±SD | False  n (%) | Partly false  n (%) | Partly true  n (%) | True  n (%) |
| --- | --- | --- | --- | --- | --- | --- |
|  | **ESI** |  |  |  |  |  |
| 1 | I speak my native language with my friends and acquaintances from my country of origin (n=326) | 3.49±0.81 | 15 (4.6) | 21 (6.4) | 79 (24.2) | 211 (64.7) |
| 2 | I have never learned to speak the language of my native country (n=326) | 1.36±0.79 | 259 (79.4) | 33 (10.1) | 19 (5.8) | 15 (4.6) |
| 3 | I eat traditional food from my native culture (n=326) | 3.33±0.86 | 20 (6.1) | 25 (7.7) | 109 (33.4) | 172 (52.8) |
| 4 | I feel comfortable speaking my native language (n=326) | 3.62±0.75 | 11 (3.4) | 20 (6.1) | 51 (15.6) | 244 (74.8) |
| 5 | I am informed about current affairs in my native country (n=326) | 3.33±0.82 | 16 (4.9) | 26 (8.0) | 118 (36.2) | 166 (50.9) |
| 6 | I know how to read and write in my native language (n=326) | 3.71±0.72 | 13 (4.0) | 14 (4.3) | 27 (8.3) | 272 (83.4) |
| 7 | I attend social functions with people from my native country (n=326) | 3.17±0.96 | 31 (9.5) | 36 (11.0) | 104 (31.9) | 155 (47.5) |
| 8 | I speak my native language at home (n=326) | 3.76±0.61 | 7 (2.1) | 10 (3.1) | 37 (11.3) | 272 (83.4) |
| 9 | I regularly read magazines of my ethnic group (n=326) | 2.13±1.14 | 140 (42.9) | 56 (17.2) | 77 (23.6) | 53 (16.3) |
| 10 | I know how to speak my native language (n=326) | 3.80±0.55 | 4 (1.2) | 11 (3.4) | 32 (9.8) | 279 (85.6) |
| 11 | I am familiar with the history of my native country (n=326) | 3.44±0.84 | 19 (5.8) | 18 (5.5) | 88 (27.0) | 210 (61.7) |
| 12 | I like to listen to music of my ethnic group (n=326) | 2.88±1.16 | 66 (20.2) | 45 (13.8) | 77 (23.6) | 138 (42.3) |
| 13 | I like to speak my native language (n=326) | 3.65±0.74 | 13 (4.0) | 13 (4.0) | 50 (15.3) | 250 (76.7) |
| 14 | I speak my native language with my spouse or partner (n=326) | 2.92±1.30 | 89 (27.3) | 22 (6.7) | 41 (12.6) | 174 (53.4) |
| 15 | When I pray, I use my native language (n=326) | 3.07±1.22 | 72 (22.1) | 17 (5.2) | 54 (16.6) | 183 (56.1) |
| 16 | I stay in close contact with my family members and relatives in my native country (n=326) | 3.45±0.90 | 23 (7.1) | 23 (7.1) | 64 (19.6) | 216 (66.3) |
|  | **DSI** |  |  |  |  |  |
| 17 | I am informed about current affairs in Hungary (n=326) | 2.63±0.89 | 48 (14.7) | 70 (21.5) | 164 (50.3) | 44 (13.5) |
| 18 | I feel totally confident with Hungarian people (n=326) | 2.50±0.98 | 61 (18.7) | 97 (29.8) | 113 (34.7) | 55 (16.9) |
| 19 | I have many Hungarian acquaintances (n=326) | 2.02±0.97 | 124 (38.0) | 102 (31.3) | 71 (21.8) | 29 (8.9) |
| 20 | I feel home in Hungary (n=326) | 2.69±0.95 | 50 (15.3) | 66 (20.2) | 146 (44.8) | 64 (19.6) |
| 21 | I feel accepted by Hungarians (n=326) | 2.62±0.98 | 51 (15.6) | 91 (27.9) | 115 (35.3) | 69 (21.2) |
| 22 | I know how to prepare Hungarian foods (n=326) | 1.90±1.02 | 157 (48.2) | 73 (22.4) | 66 (20.2) | 30 (9.2) |
| 23 | I regularly read a Hungarian newspaper (n=326) | 1.52±0.88 | 228 (69.9) | 44 (13.5) | 38 (11.7) | 16 (4.9) |
| 24 | I speak Hungarian at home (n=326) | 1.45±0.89 | 247 (75.8) | 32 (9.8) | 26 (8.0) | 21 (6.4) |
| 25 | I am familiar with important people in Hungarian history (n=326) | 2.00±0.99 | 131 (40.2) | 91 (27.9) | 76 (23.3) | 28 (8.6) |
| 26 | I think in Hungarian (n=326) | 1.36±0.81 | 263 (80.7) | 24 (7.4) | 24 (7.4) | 15 (4.6) |
| 27 | I speak Hungarian with my spouse or partner (n=326) | 1.34±0.81 | 271 (83.1) | 17 (5.2) | 21 (6.4) | 17 (5.2) |
| 28 | I like to eat Hungarian foods (n=326) | 2.59±1.09 | 73 (22.4) | 69 (21.2) | 102 (31.3) | 82 (25.2) |

Items number 1 to 16 are the questions about ethnic society immersion (ESI)

Items number 17 to 28 are the questions about dominant society immersion (DSI)
